# Cadaveric biomechanical analysis of multilevel lateral lumbar interbody fusion with and without supplemental instrumentation

**DOI:** 10.1186/s12891-021-04151-6

**Published:** 2021-03-15

**Authors:** Oujie Lai, Yunlin Chen, Qixin Chen, Yong Hu, Weihu Ma

**Affiliations:** 1 Department of Spine Surgery, Second Affiliated Hospital, Zhejiang University School of Medicine, Hangzhou, 310009 People’s Republic of China; 2 Department of Spine Surgery, Ningbo No.6 Hospital, Ningbo, 315040 People’s Republic of China

**Keywords:** Lateral lumbar interbody fusion, Biomechanics, Range of movement, Stand-alone, Lateral plate, Pedicle screw/rod

## Abstract

**Background:**

This study was to evaluate and compare the biomechanical features of multilevel lateral lumbar interbody fusion (LLIF) with or without supplemental instrumentations.

**Methods:**

Six human lumbar specimens were tested under multidirectional nondestructive moments (7.5 N·m), with a 6 degree-of-freedom spine simulator. The overall and intervertebral range of motion (ROM) were measured optoelectronically. Each specimen was tested under the following conditions at L2–5 levels: intact; stand-alone; cage supplemented with lateral plate (LP); cage supplemented with unilateral or bilateral pedicle screw/rod (UPS or BPS).

**Results:**

Compared with intact condition, the overall and intersegmental ROM were significantly reduced after multilevel stand-alone LLIF. The ROM was further reduced after using LP instrumentation. In flexion-extension (FE) and axial rotation (AR), pedicle screw/rod demonstrated greater overall ROM reduction compared to LP (*P* < 0.01), and bilateral greater than unilateral (*P* < 0.01). In lateral bending (LB), BPS demonstrated greater overall ROM reduction compared to UPS and LP (*P* < 0.01), however, UPS and LP showed similar reduction (*P* = 0.245). Intervertebral ROM reductions showed similar trend as the overall ones after using different types of instrumentation. However, at L2/3 (*P* = 0.57) and L3/4 (*P* = 0.097) levels, the intervertebral ROM reductions in AR were similar between UPS and LP.

**Conclusions:**

The overall and intervertebral stability increased significantly after multilevel LLIF with or without supplemental instrumentation. BPS provided the greatest stability, followed by UPS and LP. However, in clinical practice, less invasive adjunctive fixation methods including UPS and LP may provide sufficient biomechanical stability for multilevel LLIF.

## Background

Lumbar interbody fusion procedures are usually performed in patients with degenerative lumbar disease when conservative treatments fail to alleviate pain for prolonged period. Operative approaches to lumbar interbody fusion include posterior, transforaminal, anterior and more recently lateral transpsoas access [1, 2]. Lateral lumbar interbody fusion (LLIF) is a minimally invasive procedure, which circumvents some of the challenges and morbidity risks of anterior or posterior lumbar interbody fusion techniques, while affords necessarily indirect decompression for the treatment of spinal canal stenosis and places interbody cage without manipulation of neural structure [3]. Anterior and posterior annular/ligamentous structures can be preserved by using lateral approach, which not only limits extension motion, but tensioning of the ligaments and annulus by distraction from the lateral placed interbody cage provides substantial initial stability [4]. In addition, compared with anteriorly or posteriorly placed cages, lateral approach allows placement of larger size cages for improving bony fusion and restoring disc and foraminal heights [5, 6].

Currently, mostly biomechanical studies evaluating lateral interbody fusion were usually focused on one or two levels [4, 7, 8]. However, for patients with degenerative spinal scoliosis or multilevel lumbar disease, three or even more levels LLIF are necessary to effectively correct spinal deformity and completely decompress neural elements [9, 10]. Although, laterally placed cage was associated with superior segmental stability compared with ALIF and TLIF cages, cage subsidence and interbody un-union were usually observed in stand-alone LLIF compared to those with supplemental fixation, especially in multilevel condition [11–15]. The effect of supplemental instrumentation in multilevel LLIF procedure, such as pedicle screw/rod or lateral plate, on reducing immediately postoperative range of motion (ROM), was not elucidated clearly.

The purpose of this cadaveric biomechanical study was to evaluate biomechanical features of multilevel LLIF, and compare the stability afforded by different types of supplemental instrumentations.

## Methods

Six fresh-frozen human lumbar spine specimens (T12-L5) were used in this study (average age 51.7 years, range 31–68 years; 4 males, 2 females). Anteroposterior and lateral radiographic examinations were performed for the cadaveric specimens to exclude fracture, deformity, previous spinal surgeries, pathological disease, and other condition that could significantly affect biomechanics of spine. And dual-energy x-ray absorptionmetry scans were performed to quantify the bone mineral density (BMD) of the specimens; the average BMD was 0.935 g/cm^2^(range 0.736 g/ cm^2^–1.103 g/ cm^2^). Before testing, the specimens were thawed and stripped of the paraspinal musculature, while the discs, facet joints and osteoligamentous structures were carefully retained.

**Fig. 1:**
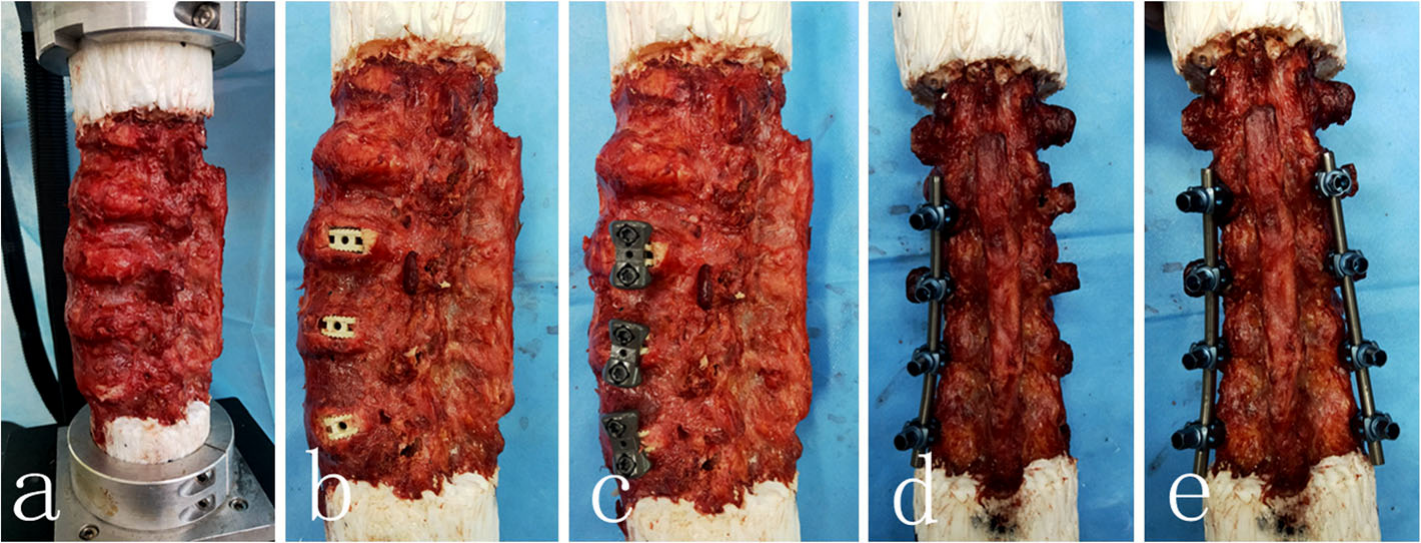
**a** Intact condition of the lumbar spine fixed at a 6 degree-of-freedom spine simulator; **b** Stand-alone construct: cages were laterally placed at L2–5 levels without instrumentation; **c** LP construct: laterally placed cages supplemented with lateral plates at L2–5 levels; **d** UPS construct: laterally placed cages supplemented with left side pedicle screw/rod at L2–5 levels; **e** BPS construct: laterally placed cages supplemented with bilateral pedicle screw/rod at L2–5 levels

In preparation for biomechanical testing, the caudal and cephalad ends of each specimen were rigidly fixed using high-strengh resin. Each testing was performed on a custom built 6 degree-of-freedom spine simulator (Fig. 1). With L3/4 level positioned horizontally, the caudal end of the specimen was rigidly fixed on the apparatus, and the cephalad end of the specimen was allowed free movement. The overall and each ROM were evaluated using an optoelectronic motion analysis system (Optotrak Certus; Northern Digital Inc., Warterloo, ON, Canada) with infrared light-emitting diode marker arrays rigidly fixed to each vertebral level, as previous described [7]. The specimens with or without supplemented instrumentation were tested under three loading conditions, including flexion-extension (FE), lateral bending (LB) and axial rotation (AR). Three loading cycles of 7.5 N·m were applied in each direction for each test condition, with the final cycle used in data analysis. These test conditions are consistent with previous literature [16].

The specimens were tested under the following condition: 1.intact condition; 2.lateral interbody cage alone (Stand-alone construct); 3.cage supplemented with lateral plate (LP construct); 4.cages supplemented with unilateral pedicle screw/rod (UPS construct); 5.cage supplemented with bilateral pedicle screw/rod (BPS construct). The specimens were initially tested in intact condition, and baseline kinematic data were recorded. After evaluation of intact spine condition, subtotal disectomy was performed from L2 to L5 level for each specimen. Discectomy include resection of ipisilateral and contralateral annulus, and removal of nucleotomy and cartilaginous endplates. Then, interbody cages (Sanyou, Shanghai) with the largest anteroposterior width of 17-mm were implanted into L2/3-L4/5 levels of each specimen. The lateral and vertical dimensions (45-55 mm in lateral and 10-15 mm in vertical) of the cages varied depending on the anatomic morphology, which had to achieve sufficient tension of ligaments and restoration of disc space height. While, to minimize cage subsidence and endplate fracture, overstuffing of the disc space should be avoided. After kinematic data collection for 3 levels stand-alone construct, laterally placed plates and screws (40 mm in length) were fixed to the three intervertebral levels. After kinematic data collection for LP construct, the lateral plates were removed. Then, the specimen was tested with unilateral and bilateral pedicle screw/rod (45 mm in length). All procedures were performed by a spine surgeon experienced with lateral interbody fusion technique.

### Statistical analysis

Statistical analysis was performed by SPSS software 22.0 (SPSS, Chicago, IL, USA). To mitigate the effect of interspecimen variability, normalization to the intact group was performed by dividing the mean ROM reduction values of different instrumented constructs with mean ROM value of the intact group. ROM reductions of different constructs relative to the intact condition were compared with a two-tailed, paired sample t test. ROM reduction comparisons between instrumentation constructs were compared with one-way analysis of variance. A significance level of *P* < 0.05 was set for all tests.

## Results

The overall ROM of L2–5 level was calculated by integration of the intervertebral value (Table 1). In intact condition, the overall ROM was 25.90 ° ± 1.26° in FE, 22.63 °± 1.66 ° in LB and 22.29 °± 0.94 ° in AR, respectively. Compared to the intact condition, any type of instrumentation constructs showed significant ROM reduction in all motion planes. Stand-alone construct reduced overall ROM by 54.58% in FE, 53.63% in LB and 31.39% in AR, respectively (*P* < 0.01). Compared to stand-alone construct, the overall ROM reduction further increased after LP fixation in all motion planes (*P* < 0.01). In FE and AR motion, pedicle screw/rod construct (bilateral and unilateral) demonstrated greater overall reduction compared to LP construct (*P* < 0.01), bilateral greater than unilateral (*P* < 0.01). In LB motion, BPS construct demonstrated greater overall reduction compared to unilateral (*P* < 0.01) and LP (*P* < 0.01) constructs, however, UPS construct failed to show significant difference compared to LP construct (*P* = 0.245).

**Table 1:** The average overall ROM of L2/3 to L4/5 levels

	Intact	Stand-alone	LP	UPS	BPS
FE	25.90 ° ± 1.26°	11.76 °± 0.62 °	8.24 °± 0.71 °	5.93 °± 0.83 °	3 °± 0.24 °
LB	22.63 °± 1.66 °	10.49 °± 0.77 °	7.97 °± 1.23 °	7.37 °± 0.81 ° ^a^	3.69 °± 0.68 °
AR	22.29 °± 0.94 °	15.29 °± 0.75 °	12.04 °± 0.72 °	9.82 °± 0.30 °	6.46 °± 0.51 °

^a^ Compared to LP, UPS failed to show significant difference in LB.

At L4/5 level, stand-alone construct reduced the ROM by 53.01% in FE, 51.71% in LB and 32.38% in AR, respectively (Table 2). LP construct reduced the ROM by 70.57% in FE (*P* < 0.01), 61.78% in LB (*P* < 0.01) and 47.51% (*P* < 0.01) in AR, respectively, showed significant improvement compared to stand-alone construct. In FE and AR motion, pedicle screw/rod construct showed greater reduction compared to LP construct (UPS vs LP: *P* = 0.018 in FE, *P* = 0.047 in AR; BPS vs LP: *P* < 0.01 in FE and AR), bilateral greater than unilateral (*P* < 0.01 in FE, *P* = 0.02 in AR). In LB motion, BPS construct demonstrated greater reduction compared to UPS (*P* < 0.01) and LP construct (*P* < 0.01). However, UPS construct failed to demonstrate greater ROM reduction in LB compared to LP construct (*P* = 0.24).

**Table 2:** The average ROM of L4/5 level

	Intact	Stand-alone	LP	UPS	BPS
FE	9.37 ° ± 0.83°	4.40 ° ±0.39 °	2.76 °± 0.32 °	2.17 ° ±0.07 °	1.15 ° ±0.17 °
LB	7.75 ° ± 0.83°	3.74 °± 0.39 °	2.96 °± 0.50 °	2.62 °± 0.41 ° ^a^	1.20 °± 0.33 °
AR	7.53 ° ±1.00 °	5.09 °± 0.44 °	3.95 °± 0.89 °	3.21 °± 0.70 °	2.31 °± 0.40 °

^a^ Compared to LP, UPS failed to show significant difference in LB.

At L3/4 level, the ROM of stand-alone construct was reduced by 55.81% in FE, 54.35% in LB and 29.28% in AR, respectively, which showed significantly less reduction than those of LP construct (*P* < 0.01) (Table 3). In FE motion, pedicle screw/rod construct demonstrated more reduction than LP construct (*P* < 0.01). In LB and AR motion, BPS demonstrated greater reduction compared with LP construct (*P* = 0.01 in LB, *P* < 0.01 in AR), whereas UPS and LP constructs afforded statistically equivalent reduction (*P* = 0.63 in LB, *P* = 0.1 in AR). BPS construct reduced the ROM by 89.01% (*P* < 0.01) in FE, 83.18% (*P* = 0.028) in LB and 72.93% (*P* < 0.01) in AR, respectively, which showed significantly greater reduction compared with UPS construct.

**Table 3:** The average ROM of L3/4 level

	Intact	Stand-alone	LP	UPS	BPS
FE	8.22 ° ± 0.80°	3.63 °± 0.64 °	2.68 °± 0.30 °	1.93 °± 0.23 °	0.90 °± 0.17 °
LB	7.30 ° ± 1.67°	3.33 °± 0.38 °	2.44 °± 0.55 °	2.22 °± 0.21 ° ^a^	1.23 °± 0.47 °
AR	7.63 ° ±0.60 °	5.40 °± 0.77 °	4.40 °± 0.54 °	3.66 °± 0.47 ° ^a^	2.07 °± 0.42 °

^a^ Compared to LP, UPS failed to show significant difference in LB and AR

At L2/3 level, the ROM of stand-alone construct was reduced by 55.07% in FE, 54.91% in LB and 32.60% in AR, respectively (Table 4). LP construct could significantly enhance the stability in all motion planes compared with stand-alone construct (*P* = 0.04 in FE, *P* < 0.01 in LB, *P* < 0.01 in AR). In FE motion, pedicle screw/rod construct showed greater reduction compared with LP construct (UPS vs LP:*P* = 0.025; BPS vs LP:*P* < 0.01). In LB and AR motion, BPS showed greater reduction compared with LP construct (*P* < 0.01), however, UPS failed to show significant reduction (*P* = 0.897 in LB; *P* = 0.057 in AR). BPS construct reduced the ROM by 88.51% (*P* = 0.044) in FE, 83.42% (*P* < 0.01) in LB and 70.79% (*P* = 0.035) in AR, respectively, which showed significantly greater reduction compared with UPS construct.

**Table 4:** The average ROM of L2/3 level

	Intact	Stand-alone	LP	UPS	BPS
FE	8.31 ° ± 0.65°	3.74 °± 0.43 °	2.81 °± 0.91 °	1.83 °± 0.67 °	0.96 °± 0.16 °
LB	7.58 ° ± 0.68°	3.42 °± 0.48 °	2.57 °± 0.54 °	2.52 °± 0.49 ° ^a^	1.26 °± 0.18 °
AR	7.14 ° ±0.84 °	4.81 °± 0.70 °	3.69 °± 0.86 °	2.95 °± 0.54 ° ^a^	2.09 °± 0.40 °

^a^ Compared to LP, UPS failed to show significant difference in LB and AR

## Discussion

LLIF technique has become an increasingly popular approach for achieving interbody fusion, allowing placement of large interbody grafts spanning the lateral borders of dense apophyseal ring bilaterally without disruption of longitudinal ligaments and posterior elements. Stand-alone interbody construction comprises a less disruptive lateral-only procedure and has also been used by some surgeons. Kretzer et al. [17] reported stand-alone cage significantly decreased ROM in all motion planes in a cadaveric study, and the addition of facet screws or pedicle screws did not show a significant improvement in stability. In a biomechanical study of L3/4 stand-alone lateral interbody fusion above a previous L4-S1 posterolateral fusion, Chioffe et al. [8] reported that the L3/4 intervertebral ROM was significantly reduced by 50% or even more in all motion planes relative to intact condition, and stand-alone LLIF was a biomechanically sound option in treatment of adjacent segment disease without necessitating revision. However, biomechanical studies evaluating multilevel LLIF were particularly limited, with most studies including one level [7, 18]. When used in multilevel procedure, whether stand-alone LLIF could provide similar stability as single level condition was doubtful. Similar to single level condition, we found multilevel stand-alone LLIF could significantly reduce the overall and intersegmental ROM in all motion planes compared with the intact condition, especially in FE and LB (> 50%). Castro et al. [12] reported that use of multilevel stand-alone LLIF for patients with degenerative scoliosis (35 cases, 107 levels) could achieved reasonable coronal and sagittal correction, as well as improvements in pain and function. However, high grade subsidence was seen in 9 patients, and 3 patients were reoperated on with pedicle screw supplementation. Watkin et al. [11] performed 37 levels of stand-alone LLIF in 22 patients, and reported the non-union incidence was 19% per level and 27% per patient. The high non-union rate was concerning for modern spine surgery. Both cage subsidence and un-union after stand-alone interbody fusion were concerning for modern spine surgery, which may cause potential risks for reoperation. Inadequate stabilization after stand-alone LLIF may allow micro-motion between the graft and endplates resulting cage subsidence and un-union.

The added instrumentation in LLIF is to further reduce motion and increase a construct’s ability to aid in fusion [19]. The optimal supplemental instrumentation for LLIF had been evaluated by many prior biomechanical and clinical studies [4, 20, 21]. In a three-dimensional finite element study of comparing the kinematic stability afforded by stand-alone cages with those supplemented by lateral plate as well as unilateral or bilateral pedicle screw/rod in a multilevel LLIF construct with simulated osteoporosis, the authors reported stand-alone cage (10–75.1% ROM reduction) and lateral plate (23.9–86.2% ROM reduction) provided inadequate ROM restriction for the multilevel LLIF constructs, whereas lateral cage with BPS (66.7–90.9% ROM reduction) or UPS (45.0–88.3% ROM reduction) fixation provided favorable biomechanical stability [13]. Nayak et al. [7] compared the acute biomechanically stabilizing effects of the two-hole lateral plate and BPS constructs in lumbar spine instrumented with lateral cage at L3/4 and L4/5 levels. The ROM at L3/4 level was reduced by 82.6% in FE, 89.8% in LB, and 53.4% in AR for BPS construct, compared with 52.3, 71 and 45.3% for LP construct, respectively. The ROM at L4/5 level was reduced by 88.9% in FE, 93.2%% in LB, and 69.8% in AR for BPS construct, compared with 47.1, 64.5 and 50.4% for LP construct, respectively. Each intervertebral ROM at the instrumented level was significantly reduced in any motion planes relative to the intact condition, and BPS construct afforded the greatest ROM reduction.

In this multilevel lateral biomechanical study, each type of instrumentation constructs could effectively enhance the overall and intervertebral stability in all loading modes compared with the stand-alone construct. Among all types of instrumentation constructs, BPS construct provided the greatest overall and intervertebral ROM reduction, followed by UPS, as well as LP. While, the overall or each intervertebral lateral motion reduction (> 60%) was statistical comparable between LP and UPS constructs. Cappuccino et al. [4] reported that LP construct could reduce lateral motion more than 80%, which showed no significance compared with BPS construct. Fogel et al. [22] also reported addition of a lateral plate to the interbody cage could significantly decrease the ROM in LB, and demonstrated no significance compared with BPS construct. These results demonstrated LP instrumentation could effectively reduce lateral motion in LLIF procedure. There is controversy regarding the effect of LP on AR reduction. Comparable AR reduction between LP and BPS constructs was reported by Nayak et al. [7]. In the study of Cappuccino et al. [4], ROM reduction in AR was not significantly different between UPS and LP constructs, whereas BPS construct exhibited significantly greater ROM reduction than LP and UPS construct. In our study, there was comparable ROM reduction in AR at L2/3 and L3/4 levels between LP and UPS constructs, however, UPS construct exhibited significantly greater overall and L4/5 intersegmental ROM reduction than LP construct. This discrepancy may arise from different testing methodology, measuring device, instrumentation and cadaver specimens.

In clinical practice, both minimal tissue damage and sufficient stability are important factors for optimal postoperative results. BPS instrumentation provided the greatest stability in any motion plane, however, the tissue damage caused was also the largest. Katz et al. [9] performed LLIF with posterior instrumentation to treat 3 or even more contiguous levels of degenerative lumbar scoliosis. During a period of 22.5 months follow-up, the patients showed significant clinical and radiographic improvement, however 37% patients experienced complications, which was significantly correlated with open-posterior portion rather than the number of LLIF levels treated. Wen et al. [23] evaluated the outcomes of percutaneous UPS and BPS fixations after single-level oblique lateral interbody fusion procedures (OLIF). Their study showed OLIF with UPS fixation resulted in less blood loss and operative time, while, had comparable effects on radiological and clinical outcomes to that of BPS fixation. In a study of using OLIF combined with anterolateral screw fixation for the treatment of lumbar degenerative disc disease, the incidence of cage subsidence was only 7.7%, and no revision was performed due to biomechanical failure [24]. The authors reported it was a relatively safe and effective surgical option for lumbar degenerative disc disease. Dakwar et al. [25] also reported the treatment strategy of lateral plate combined with multilevel lateral interbody fusion for adult degenerative scoliosis was associated with minimal invasiveness and excellent biomechanical stability. Because of the inherent stability of lateral implanted large sized cage and minimal disruption of stabilizing ligaments, less invasive adjunctive fixation methods including UPS and LP may allowed to use in multilevel LLIF, even which afford less biomechanical stability compared with BPS.

This study had several potential limitations. The group in this study was a small sample size, limiting its statistical power. Simplified loading was applied to be repeatable between specimens and independent of specimen size. Standardly sized pedicle screw and lateral screw were used in each level and each specimen in this study. Furthermore, the results reported in this study described the immediately postoperative stability of different types of constructs, which could not truly reflect biological changes occurring in vivo. Finally, all musculature was removed from the specimen, hence, the influence of paraspinal muscle on construct was neglected during biomechanical analysis.

### Conclusions

In conclusion, the results of this biomechanical study indicated stand-alone, LP, UPS or BPS constructs could significantly reduce the overall and intervertebral ROM after multilevel LLIF. BPS provided the greatest stability, followed by UPS and LP constructs. In clinical practice, less invasive adjunctive fixation methods including UPS and LP may provide sufficient biomechanical stability for multilevel LLIF, even which afford less biomechanical stability compared with BPS.

## Data Availability

The datasets used and/or analyzed during the current study are available from the corresponding author on reasonable request.
